# Dreaming, Mind-Wandering, and Hypnotic Dreams

**DOI:** 10.3389/fneur.2020.565673

**Published:** 2020-10-09

**Authors:** Peter Fazekas, Georgina Nemeth

**Affiliations:** ^1^Center of Functionally Integrative Neuroscience, Aarhus University, Aarhus, Denmark; ^2^Centre for Philosophical Psychology, University of Antwerp, Antwerp, Belgium

**Keywords:** dreaming, mind-wandering, hypnosis, hypnotic dreams, hypnotic analog

## Abstract

Hobson's AIM theory offers a general framework for thinking about states of consciousness like wakefulness, REM dreaming and NREM mentations in terms of a state space defined by the dimensions of the level of brain activity, the source of input, and the type of neurochemical modulation. This account inspired theoretical models of other altered states of consciousness—including hypnosis—claiming that studying REM dreaming can advance our understanding of these phenomena as well. However, recent developments showed that hypnosis is not a sleep like stage, and that the REM-centric attitude toward dreaming is mistaken. At the same time, the advancement of the neuro-cognitive theory claiming that dreaming and mind-wandering are on a continuum both underlain by default-mode network activity called many aspects of the AIM theory into question. Our aim in this paper is to show that certain hypnotic states—hypnotic dreams (experiences that subjects have in a hypnotic state as a result of an explicit suggestion to have a dream)—can, nevertheless, be highly relevant for the neuro-cognitive theory, and that their comparison with dreaming and mind-wandering has the potential to advance the field in unexpected ways.

## Introduction

Allan Hobson's Activaton-Input-Modulation (AIM) theory of dreaming claiming that REM dreaming and NREM mentations are subserved by different mechanisms and can best be understood in terms of a state space defined by the dimensions of the level of global brain activity (low-high), the source of input (external-internal), and the type of neurochemical modulation (aminergic-cholinergic) has been extremely influential not just in dream science (1), but also in studying hallucinations (2) and understanding other altered states of consciousness, like lucid-dreaming (3) and also hypnosis (4). The traits of absorption and fantasy proneness are strongly associated with hypnotic susceptibility [the depth of trance individuals are able to reach; see (5, 6)], and are also key characteristics of dreaming (4) correlating with its vividness and with dream recall frequency (7). This, together with certain phenomenological similarities with regard to e.g., diminished responsiveness to external stimuli, enhanced hallucinations, occasionally impaired orientation, suspended volition, and enhanced memory for past events inspired Hobson et al. to theorize that studying rapid eye movement (REM) sleep and dreaming is able to shed new light on hypnosis (4).

However, developments in hypnosis research have demonstrated that hypnosis is not a sleep-like state (8), and the REM-centric attitude toward dreaming that the AIM model relied on has been severely criticized over the years on several grounds (9–13). With the advancement of the rival neuro-cognitive theory of dreaming stating that all forms of dreaming and mind-wandering are on a continuum underlain by default-mode network activity (10, 14), the unificatory view that presented dreaming and hypnosis in analogy with each other has become obsolete. As Domhoff argues, the purported parallel between dreams and hypnotic states is “extremely misleading” and should be ignored (10).

Despite this strong resistance, our aim in this paper is to show that certain hypnotic states can, in fact, be highly relevant for the neuro-cognitive theory, and that their comparison with dreaming has the potential to advance the field in unexpected ways.

## Hypnotic Dreams

The hypnotic states in question are so-called *hypnotic dreams*—experiences that subjects have in a hypnotic state as a result of an explicit suggestion to have a dream. They are often used in clinical settings as a tool during hypnotherapy (15, 16), and even feature as an item in the clinical scales measuring hypnotic responsiveness (17, 18). Yet, from the perspective of cognitive psychology and neuroscience, they are extremely under-explored.

Hypnotic dreams can be induced via content-neutral suggestions, i.e., without a specific instruction about what the dream should be about. Early studies during the middle of the previous century reported contradictory findings with regard to whether hypnotic dreams showed phenomenological similarities with nocturnal dreams (19, 20) or with daydreams (21, 22). It has been argued that hypnotic dreams form a broad category with different types like simply thinking about something; mind-wandering (daydreaming); vivid hallucinations like watching a film; and feeling located in a dream world (23). This variance in how dream-like a hypnotic dream appeared to be had been associated with the depth of hypnosis, with deeper levels of trance (the altered state of consciousness experienced during hypnosis deeper levels of which occur in individuals with higher hypnotic susceptibility) resulting in more symbolic and more dream-like hypnotic dreams (19).

In a study specifically targeting this question, Barrett (24) collected 285 hypnotic dreams, 285 daydreams, and 277 nocturnal dreams, and analyzed their content and formal characteristics (15, 24). Barrett found that in their content and formal characteristics hypnotic dreams in deep trance were similar of dreaming, while in medium trance (occurring in individuals with medium hypnotic susceptibility) they were similar to mind-wandering (15, 16, 24).

Parallel investigations focusing on the physiological level have been extremely rare. Some early studies found that hypnotic dreams in deep-trance were accompanied by eye movements that were indistinguishable from REMs (24–26). Although the relevance of this finding has decreased in the light of the dissociation of dream experiences from the REM stage of sleep, it still might be indicative of at least a certain degree of physiological similarity between hypnotic and nocturnal dreams. The only neural level study published so far (a very coarse grained analysis with only 4 EEG channels) gestures toward the same direction (27). It reports increased 40 Hz activity during deep-trance hypnotic dreams (compared to a rest in hypnosis condition) over the right posterior (especially parietal) areas, which is roughly in line with the recent claim that increased high-frequency activity of posterior content-specific regions is the neural correlate of the content of dream, lucid dreaming and mind-wandering experiences (28–31).

## The Hypnotic Analog Relation

On the basis of these similarities, it is an interesting question whether hypnotic dreams are in a hypnotic analog relation with nocturnal dreams and mind-wandering. Instrumentalist hypnosis research uses hypnosis as a tool for exploring other psychological processes and phenomena by inducing and modulating specific cognitive and perceptual states (32). Such a cognitive or perceptual state occurring in hypnosis is a hypnotic analog of a target phenomenon if they have a high degree of similarity in phenomenological characteristics and significant overlaps in correlating neural activity patterns. In instrumentals practice, first a hypnotic analog of a phenomenon is established on the basis of these phenomenological similarities and neural overlaps, and then hypothesis-driven studies use it to test predictions regarding the specific target phenomenon in question (33–35). The main benefit of this methodology is that it can offer a degree of control over intractable phenomena that otherwise wouldn't be possible.

The last two decades have seen a proliferation of instrumentalist hypnosis research with regard to phenomena like hallucinations (36), clinical delusions (37–39), clinical confabulation (40), and functional pain [which is experienced in the absence of any internal or external stimulus or nerve damage; (41)]. In the case of functional pain, for example, the hypnotic analog relation between heat induced pain and the hypnotically induced analog was established by a detailed comparison both at the level of subjective experiences and fMRI-measured brain activity (41, 42). A significant overlap was found in the brain area that showed activity during heat induced pain. Both in hypnotically and in heat induced pain there was a direct relationship between the reported intensity of the pain and the degree of activation of pain areas, with the activity of the overlapping brain regions contributing similarly to the subjective feel of pain.

## Discussion

Unfortunately, empirical data about hypnotic dreams at this level of detail—especially about their neural underpinnings—are not available. Therefore, evaluating whether a hypnotic analog relation could be established between hypnotic and nocturnal dreams is currently out of reach. To make such an evaluation possible future studies are required to explore the similarities and differences in the neural substrate of these mental states. In the rest of the paper we will focus on theoretical considerations that will shed new light on why hypnotic dreams might be relevant for our understanding of dreaming and its relation to mind-wandering.

A central tenet of the neuro-cognitive theory of dreaming is the so-called continuity hypothesis that asserts that dream cognition is similar to waking cognition—i.e., the same conceptions and concerns are the basis for dreaming, waking thoughts, and waking actions. In this framework, dreaming is interpreted as an intensified form of mind-wandering (10, 43, 44). According to a highly influential characterization (45), mind-wandering is essentially spontaneous: during these episodes thoughts and imagery arise and unfold in a relatively free, unconstrained way [for an opposing view see (46)].

From this point of view, turning toward hypnotic dreams to advance our understanding of dreaming and mind-wandering might seem ill-advised. There is a general consensus that spontaneously occurring thoughts are reduced in the hypnotic state (33, 47, 48), and that hypnotic induction leads to a decreased activity in the default mode network (34), whereas both dreaming and mind-wandering are associated with increased default-mode network activity (43, 49).

However, hypnotic dreams are in an important respect quite unlike typical hypnotic phenomena: the specific suggestions initiating them are neutral with regard to the content of the experience. Hence the content of hypnotic dreams develops spontaneously, independently of cognitive control processes, without any constraints imposed either by the hypnotist or by volition on the subject's part. Indeed, studies focusing on neutral hypnosis (in which no explicit suggestions are administered during induction other than to become hypnotized) and self-hypnosis (in which the hypnotic state is self-induced) show that they involve increased spontaneous imagery, free-floating attention and receptivity to internal stimuli (50, 51). Furthermore, the tension between the association of dreaming and mind-wandering with increased default mode network activity and the hypnosis-specific findings to the contrary might be resolved by the fact that in the case of hypnosis, decreased default mode network activity is a consequence of hypnotic induction, i.e., a characteristic of the brain entering the general hypnotic state, which then, based on the specific suggestions can change (might even re-increase) significantly (48).

These considerations allow us to go beyond the proposal that high trance hypnotic dreams are similar to nocturnal dreams whereas medium trance hypnotic dreams are similar to episodes of mind-wandering. From the perspective of the dynamics of the experience, medium trance hypnotic dreams induced by a content-neutral “have a dream” suggestion are like purposefully initiated—i.e., intentional (52)—instances of mind-wandering. To see why, consider that the depth of hypnotic trance positively correlates with the deactivation of cognitive control processes (33, 34) and that cognitive control involvement in intentional mind-wandering is greater than in unintentional mind-wandering (52). Given all this, the fact that in hypnotic dreams cognitive control plays a role only in the initiation and maintenance of the hypnotic dream experience (but not in determining its content, due to the content-neutral nature of the corresponding suggestions) leads to the hypothesis that hypnotic dreams at a level of trance closer to the lower end of the medium range might be more similar to intentional, while those at a level of trance closer to the higher end of the medium range might be more similar to unintentional mind-wandering.

Even if future research will not be able to establish a hypnotic analog relation, studying hypnotic dreams has the potential to provide unexpected support for the neuro-cognitive theory of dreaming by demonstrating of a phenomenon—hypnotic dreams—that originally fell outside of the scope of the neuro-cognitive theory that it is on the same continuum that is defined by mind-wandering and dreaming.

## Data Availability Statement

All datasets presented in this study are included in the article/supplementary material.

## Author Contributions

PF and GN together planned and invented the paper's argumentation. PF wrote the manuscript, GN reviewed it, then PF and GN together created the final version. All authors contributed to the article and approved the submitted version.

## Conflict of Interest

The authors declare that the research was conducted in the absence of any commercial or financial relationships that could be construed as a potential conflict of interest.
